# Safety of conscious sedation in electroanatomical mapping procedures and cryoballoon pulmonary vein isolation

**DOI:** 10.1007/s00380-020-01725-7

**Published:** 2020-11-19

**Authors:** Karolina Weinmann, Regina Heudorfer, Alexia Lenz, Deniz Aktolga, Manuel Rattka, Carlo Bothner, Alexander Pott, Wolfgang Öchsner, Wolfgang Rottbauer, Tillman Dahme

**Affiliations:** 1 Department of Internal Medicine II, Ulm University Medical Center, Albert-Einstein-Allee 23, Ulm, Germany; 2 Department of Anesthesiology, Ulm University Medical Center, Ulm, Germany

**Keywords:** Conscious sedation, Electrophysiology, Electroanatomical mapping, Cryoballoon pulmonary vein isolation, Sedation safety

## Abstract

Immobilization of patients during electrophysiological procedures, to avoid complications by patients’ unexpected bodily motion, is achieved by moderate to deep conscious sedation using benzodiazepines and propofol for sedation and opioids for analgesia. Our aim was to compare respiratory and hemodynamic safety endpoints of cryoballoon pulmonary vein isolation (PVI) and electroanatomical mapping (EAM) procedures. Included patients underwent either cryoballoon PVI or EAM procedures. Sedation monitoring included non-invasive blood pressure measurements, transcutaneous oxygen saturation (tSpO_2_) and transcutaneous carbon-dioxide (tpCO_2_) measurements. We enrolled 125 consecutive patients, 67 patients underwent cryoballoon atrial fibrillation ablation and 58 patients had an EAM and radiofrequency ablation procedure. Mean procedure duration of EAM procedures was significantly longer (*p* < 0.001) and propofol doses as well as morphine equivalent doses of administered opioids were significantly higher in EAM patients compared to cryoballoon patients (*p* < 0.001). Cryoballoon patients display higher tpCO_2_ levels compared to EAM patients at 30 min (cryoballoon: 51.1 ± 7.0 mmHg vs. EAM: 48.6 ± 6.2 mmHg, *p* = 0.009) and at 60 min (cryoballoon: 51.4 ± 7.3 mmHg vs. EAM: 48.9 ± 6.6 mmHg, *p* = 0.07) procedure duration. Mean arterial pressure was significantly higher after 60 min (cryoballoon: 84.7 ± 16.7 mmHg vs. EAM: 76.7 ± 13.3 mmHg, *p* = 0.017) in cryoballoon PVI compared to EAM procedures. Regarding respiratory and hemodynamic safety endpoints, no significant difference was detected regarding hypercapnia, hypoxia and episodes of hypotension. Despite longer procedure duration and deeper sedation requirement, conscious sedation in EAM procedures appears to be as safe as conscious sedation in cryoballoon ablation procedures regarding hemodynamic and respiratory safety endpoints.

## Introduction

The number of complex electrophysiological procedures has increased over the last decades [1, 2]. For treatment of atrial arrhythmia, different techniques dependent on the type of arrhythmia can be applied: pulmonary vein isolation (PVI) to treat atrial fibrillation may be achieved either by point-by-point radiofrequency ablation guided by 3D-mapping systems or by single-shot anatomical ablation devices, such as the cryoballoon [3]. Regular atrial tachyarrhythmia, i.e. macroreentrant, microreentrant or focal atrial tachycardia, require electroanatomical mapping and therefore generally are treated by 3D-mapping guided point-by-point radiofrequency catheter ablation [1]. Most electrophysiological procedures can be performed under conscious moderate to deep sedation [4–6]. Immobilization of patients during long-lasting procedures is required to avoid complications by patients’ unexpected bodily motion [7]. A common approach to deliver moderate-to-deep conscious sedation is a proceduralist-directed nurse-administrated (PDNA) model, using benzodiazepines and propofol for sedation and opioids for analgesia [5, 8]. Administered medication has depressive effects on the central nervous system, especially the respiratory center and on the cardiovascular system. Mandatory monitoring includes continuous oxygen saturation by pulse oximetry, non-invasive or invasive blood pressure measurements and continuous electrocardiographic monitoring [4]. Arterial or venous blood gas analysis for carbon-dioxide partial-pressure monitoring may add additional safety by preventing hypercapnia. Continuous, transcutaneous carbon-dioxide measurement may provide more detailed, real-time information on the ventilatory status of the patient without the necessity to draw and analyze blood samples. At our center, depending on the sedation requirements of different techniques, we apply differential sedation schemes for cryoballoon procedures and 3D-mapping procedures. 3D-mapping requires a strictly immobilized patient to avoid map shifts and procedure associated complications [9]. In contrast, for cryoballoon ablation, moderate conscious sedation is usually sufficient. Cryoballoon ablation has been shown to be at least equally effective and equally safe to treat atrial fibrillation as point-by-point radiofrequency atrial fibrillation ablation [3, 10]. So far, there is a gap of evidence regarding the safety of deep conscious sedation required for 3D electroanatomical mapping in comparison to moderate conscious sedation for cryoballoon procedures.

## Materials and methods

### Study population

In this non-randomized, prospective observational study, consecutive patients undergoing electroanatomical mapping procedures or cryoballoon PVI ablation under deep or conscious sedation with additional transcutaneous carbon-dioxide partial-pressure (tpCO_2_) monitoring were considered for enrollment and analysis from January 2019 to August 2019 in Ulm University medical center.

### Sedation management and monitoring protocol

At the beginning of the procedure, the patients receive in general 5 mg midazolam bolus and simultaneously a continuous propofol (20 mg/ml) administration by perfusor is installed. The depth of sedation was monitored clinically by the assisting nurse and propofol infusion rate was titrated to the optimum sedation depth required for the examination. An additional administration of opiates (fentanyl bolus 50 µg in cryoballoon AF ablation or continuous remifentanil for electroanatomical mapping procedures) was performed before transseptal puncture and ablation. The airway was maintained by a guedel airway and if necessary patients received oxygen supply via mask. The patients’ respiratory and hemodynamic monitoring included continuous measurement of heart rate, continuous electrocardiogram and pulsoxymetric oxygen saturation (SpO_2_). Every 3 min, a non-invasive blood pressure measurement was performed and half hourly an analysis of venous blood gas was performed. All enrolled patients received an additional, continuous tpCO_2_ monitoring using TCM 400 (Radiometer America™, Westlake, OH). The tpCO_2_-sensor was positioned at the forehead of every patient scheduled for electrophysiological procedure in deep or conscious sedation. Sedative medication was administrated, when the tpCO_2_-monitoring displayed a stable waveform after self-calibration. The TCM 400 records an Excel® protocol documenting every two seconds, time, tpCO_2_, power, sensor temperature, transcutaneous oxygen saturation (tSpO_2_) and heart rate. At the end of examination and after an adequate awaken patient, the sensor on the forehead is removed. Time point and dose of administrated medication, actions regarding airway management (i.e. guedel airway, oxygen administration) and results of blood gas analysis are documented in the sedation protocol by the nurse that is assisting the sedation.

### Cryoballoon pulmonary vein isolation

A 6F steerable decapolar catheter was placed in the coronary sinus (CS). Left atrial (LA) access was obtained by a single transseptal puncture under fluoroscopy guidance using a Brockenbrough catheter and a 2H transseptal needle (Maslanka, Tuttlingen, Germany). After administrating a heparin bolus, aiming an activated clotting time of > 300 s, a guidewire was advanced in the left superior PV and a 12F steerable sheath (Flexcath advance, Medtronic, USA) was positioned in the left LA. All PV ostia were detected by PV angiography followed by introduction of a 28 mm cryoballoon (Arctic Front Advance Pro, Medtronic, USA) in the LA and guided to the target PV over a 20-mm spiral mapping catheter (Achieve, Medtronic). Ablation was performed according to an individualized time-to-isolation protocol. The esophageal temperature was monitored by a nasally placed temperature probe (Sensitherm; St. Jude Medical Inc, St Paul, MN, USA or S-Cath; Circa Scientific Inc., USA) in the esophagus, at the closest possible proximity to the ablation site. A luminal esophageal temperature of 15–20 °C was the cut-off temperature, leading to abortion of the freeze cycle. Phrenic nerve function was monitored by phrenic nerve stimulation and detection of compound motor action potentials (CMAP), as well palpation of diaphragm contractions during ablation of the right sided PVs [11, 12].

### Electroanatomical mapping procedure

Mapping procedure ablation sites included mainly left atrial but also right and biatrial mapping procedures. Despite the varying ablation sites, electroanatomic mapping procedures are highly standardized at our center. Access to the right ventricle is achieved by the right femoral vein and a 6F steerable decapolar catheter was placed in the coronary sinus (CS) as reference. If necessary, left atrial (LA) access was obtained by a double transseptal puncture equivalent to the cryoballoon PVI procedure. An 8.5F steerable sheath (Agilis NXT, Abbott Inc, St. Paul, MN) and an 8.5F non-steerable sheath (SL1, Abbott Inc, St. Paul, MN) were positioned in the left LA. A PV angiography is performed. For electroanatomic mapping approaches, ENSITE™ Precision (Abott®, Abbott Inc, St. Paul, MN) and CARTO® 3 System (Biosense Webster®, Irvine, California, USA) mapping systems are used. For PV evaluation, a 20-polar spiral mapping catheter (Advisor™ Abbott Inc, St. Paul, MN or Lasso®, Biosense Webster, Diamond Bar, CA, USA) was used, for high-resolution mapping a PentaRay® (Biosense Webster, Diamond Bar, CA, USA) or Advisor™ HD Grid (Abbott Inc, St. Paul, MN) were used. Additionally, an irrigated-tip ablation catheter with contact-force sensing was used (TactiCath™, Abbott Inc, St. Paul, MN; Thermocool Smarttouch® SF, Biosense Webster, Diamond Bar, CA, USA).

### Statistical analysis

Statistical analyses were performed using GraphPad Prism 6 Statistics® (GraphPad Software, Inc., CA 92037, USA). Groups were compared by two-sided Mann–Whitney test, a *p* value < 0.05 was considered statistically significant.

### Ethics

The investigation conforms with the principles outlined in the Declaration of Helsinki. The device and detector for tpCO_2_ monitoring have completed CE-certification and FDA approval and are non-investigational. Written informed consent was obtained from each patient and the protocol was approved by the Ethics Committee of the University of Ulm under the official designation number 324/16.

## Results

### Baseline characteristics of the study population

**Fig. 1:**
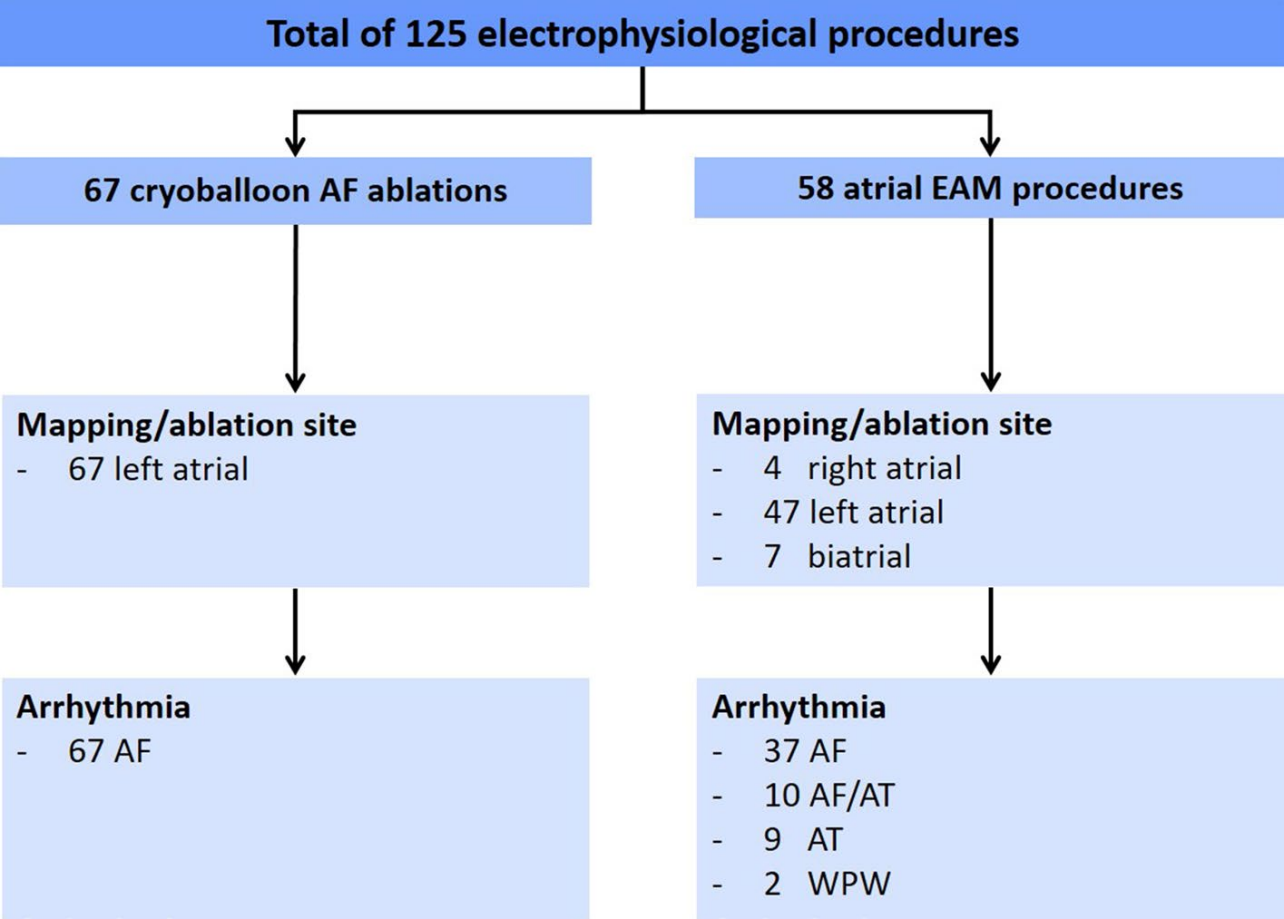
Mapping/ablation site and arrhythmia. Detailed overview of the included electroanatomical mapping and cryoballoon procedures, including mapping/ablation site and regarding arrhythmia. *AF* atrial fibrillation, *AT* atria tachycardia, *EAM* electroanatomical mapping, *WPW* wolff parkinson white syndrome

In total, 125 patients were included in the analysis. 67 patients underwent cryoballoon pulmonary vein isolation (PVI) for atrial fibrillation and 58 patients had an atrial electroanatomic mapping (EAM) and radiofrequency (RF) ablation procedure. Detailed information on ablation site and arrhythmia is provided in Fig. 1. The mean age of the patients was 66.8 ± 12.3 years and was similar in the cryoballoon PVI and EAM group and 73 patients (58%) of the total cohort were male and 52 patients (42%) were female. The distribution between female and male gender was similar in the cryoballoon PVI and the EAM group (*p* = 0.96). The proportion of obese patients did not differ between groups. Only few patients of the total cohort had pulmonary diseases such as COPD, asthma or sleep apnea, and were distributed equally to the cryoballoon PVI and the EAM group. Liver function was assessed by AST and γGT, renal function by creatinine, glomerular filtration rate and chronic kidney disease stadium. Renal and liver function did not differ between both groups. Detailed information of all baseline characteristics is provided in Table 1.

**Table 1:** Baseline characteristics

	Total	Cryoballoon PVI	3D-mapping	*p* value
Patients, *n*	125	67	58	
Age, years	66.8 ± 12.3	66.6 ± 12.1	67.0 ± 12.5	0.84
Male, *n* (%)	73 (58)	39 (58)	34 (59)	0.96
BMI (Body mass index), kg/m^2^	28.5 ± 6.0	28.6 ± .5.7	28.5 ± 6.2	0.96
Obesity (BMI ≥ 30 kg/m^2^), *n* (%)	32 (26)	20 (30)	12 (21)	0.33
Normal LVEF, *n* (%)	91 (73)	46 (69)	45 (78)	0.36
Coronary artery disease, *n* (%)	49 (39)	28 (42)	21 (36)	0.65
Hypertension, *n* (%)	91 (73)	50 (75)	41 (71)	0.77
Diabetes mellitus, *n* (%)	31 (25)	18 (27)	13 (22)	0.71
Smoker, *n* (%)	6 (5)	5 (7)	1 (2)	0.21
Former-Smoker, *n* (%)	28 (22)	14 (21)	14 (24)	0.83
Pulmonary disease				
COPD, *n* (%)	4 (3)	2 (3)	2 (2)	1.00
Asthma, *n* (%)	4 (3)	1 (2)	3 (5)	0.34
Sleep apnea, *n* (%)	4 (3)	3 (4)	1 (2)	0.62
Liver function				
AST, (U/L)	25.2 ± 7.7	25.2 ± 8.0	25.3 ± 7.3	0.97
γGT, (U/L)	48.1 ± 40.1	46.2 ± 31.0	50.3 ± 48.6	0.57
Renal function				
Creatinine,, (µmol/L)	99.7 ± 46.6	100.1 ± 57.7	99.2 ± 28.9	0.91
GFR, (mL/min)	67.4 ± 22.4	68.8 ± 22.0	65.9 ± 22.8	0.48
CKD ≥ 2, *n* (%)	105 (84)	57 (85)	48 (83)	0.91

Values are *n*, *n* (%), mean ± SD

*SD* standard deviation, *LVEF* left ventricular ejection fraction, *GFR* glomerular filtration rate, *CKD* chronic kidney disease

### Longer procedure duration and higher propofol use in EAM procedures with the same safety level

Comparison of the procedure duration of cryoballoon PVI and EAM procedures showed a significant difference (*p* < 0.001). Mean duration of EAM procedures was twice as long as a cryoballoon PVI (159.9 ± 53.0 min vs. 96.3 ± 34.5 min). The total midazolam use is not different between groups (cryoballoon: 4.9 ± 0.8 mgvs. EAM: 4.9 ± 0.4 mg, *p* = 0.81). The sedation is maintained by continuous propofol administration, thus it is not surprising that the mean dose of administered propofol in EAM procedures is significantly higher than in cryoballoon PVI (681.0 ± 350.5 mg vs. 484.5 ± 200.9 mg, *p* < 0.001). A mean dose of 189 ± 92 µg remifentanil was administered continuously in EAM procedures and a mean dose of 74.6 ± 32.8 µg fentanyl was administered in boli in cryoballoon PVI procedures. Comparison of the morphine equivalent dose shows that EAM patients received a significantly higher doses than cryoballoon PVI patients (35.3 ± 20.2 mg vs. 9.0 ± 3.9 mg, *p* < 0.001) (Table 2).

**Table 2:** Procedural characteristics

	Total	Cryoballoon PVI	EAM	*p* value
Procedure duration, minutes	125.8 ± 54.3	96.3 ± 34.5	159.9 ± 53.0	** < 0.001**
Sedative medication				
Midazolam, mg	4.9 ± 0.6	4.9 ± 0.8	4.9 ± 0.4	0.81
Propofol, mg	575.7 ± 297.0	484.5 ± 200.9	681.0 ± 350.5	** < 0.001**
Fentanyl, µg	74.6 ± 32.8	74.6 ± 32.8	–	
Remifentanyl, µg	189 ± 92	–	189 ± 92	
Morphine equivalent dose, mg	44.2 ± 24.1	9.0 ± 3.9	35.3 ± 20. 2	** < 0.001**

Bold values indicate statistically significant *p* values

Values are *n*, *n* (%), mean ± SD

*EAM* electroanatomical mapping, *MAP* mean arterial pressure, *SD* standard deviation

### Time course of hemodynamic and respiratory parameters

**Fig. 2:**
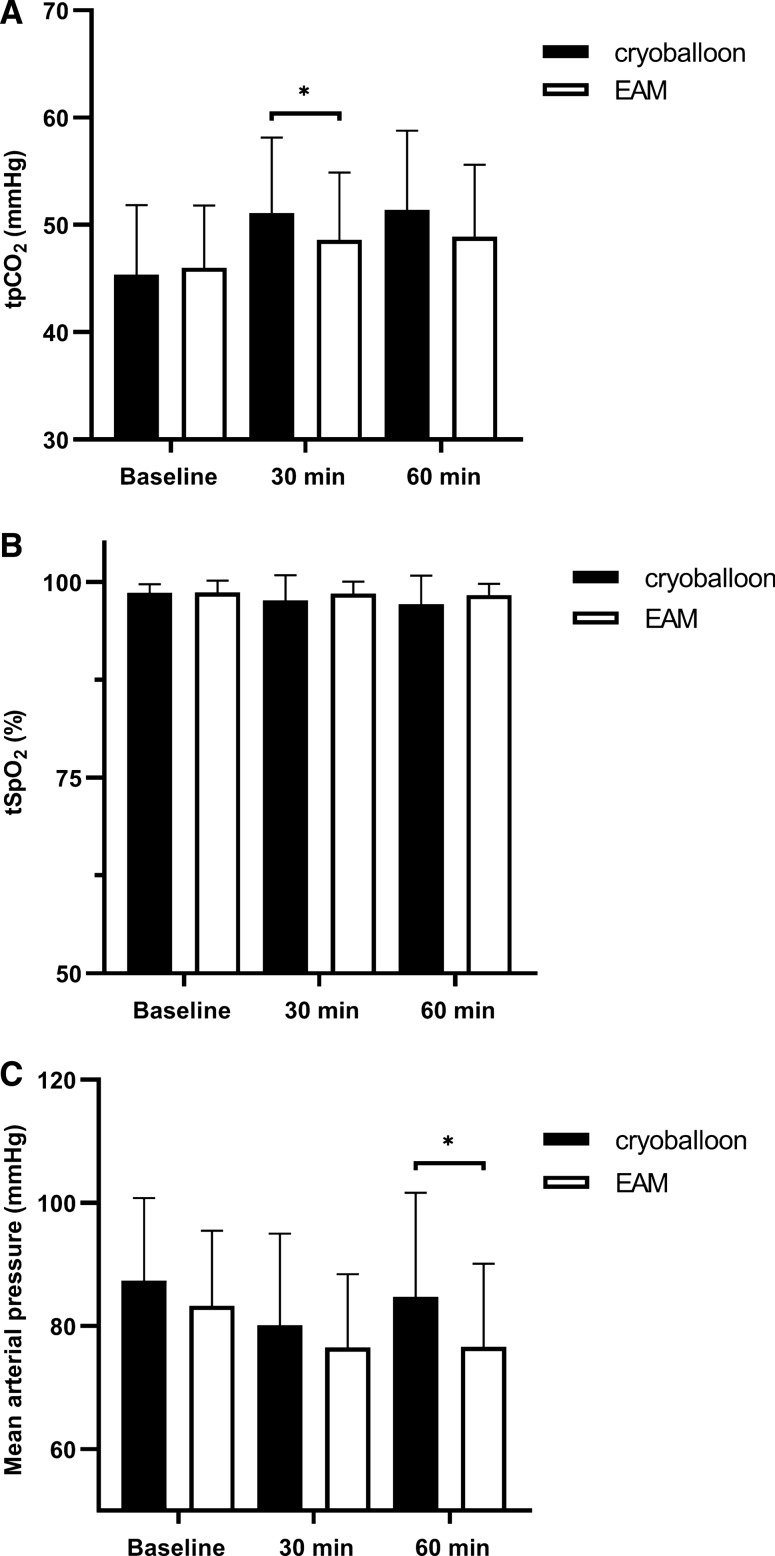
Respiratory and hemodynamic parameters. **a** Mean values of TpCO_2_ are increased in the cryoballoon group after 30 min (*p* = 0.009) and 60 min (*p* = 0.07) compared to the EAM group. **b** TpO2 values are slightly lower in cryoballoon patients after 30 min (*p* = 0.08) and 60 min (*p* = 0.19). **c** Mean arterial pressure (MAP), does not differ after 30 min (*p* = 0.29) between groups, but is significantly different after 60 min (*p* = 0.017) in cryoballoon ablation procedures compared to EAM procedures. *EAM*, electroanatomical mapping

Hemodynamic and respiratory monitoring includes half-hourly venous blood gas analysis, continuous transcutaneous monitoring and non-invasive blood-pressure measurements. Transcutaneous pCO_2_ values are similar at baseline, but show a significant difference at 30 min (cryoballoon: 51.1 ± 7.0 mmHg vs. EAM: 48.6 ± 6.2 mmHg, *p* = 0.009) and trend at 60 min (cryoballoon: 51.4 ± 7.3 mmHg vs. EAM: 48.9 ± 6.6 mmHg, *p* = 0.07). Cryoballoon patients display a higher tpCO_2_ level compared to EAM patients. However, in both groups, mean tpCO_2_ levels were within the reference range throughout the procedure. Parallel to the higher tpCO_2_ the tSpO_2_ levels were slightly lower in cryoballoon procedure patients compared to EAM patients at 30 min (cryoballoon: 97.7 ± 3.2% vs. EAM: 98.5 ± 1.5%, *p* = 0.08) and 60 min (cryoballoon: 97.1 ± 3.6% vs. EAM: 98.3 ± 1.4%, *p* = 0.19) time point. Mean arterial pressure (MAP), measured non-invasively, does not differ between groups after 30 min procedure duration (cryoballoon: 80.1 ± 14.8 mmHg vs. EAM: 76.5 ± 11.8 mmHg, *p* = 0.29). After 60 min, MAP is significantly higher (cryoballoon: 84.7 ± 16.7 mmHg vs. EAM: 76.7 ± 13.3 mmHg, *p* = 0.017) in cryoballoon ablation procedures compared to EAM procedures (Fig. 2).

### Electroanatomical mapping procedures and cryoballoon ablation procedures are similar in hemodynamic and respiratory safety endpoints

Continuous, transcutaneous monitoring and non-invasive BP measurements provide detailed information on respiratory and hemodynamic endpoints. Hypotension, with a MAP of < 65 mmHg, hypercapnic episodes (tpCO_2_ > 70 mmHg), hypoxic episodes (tpO_2_ < 90%) and cumulative time of hypoxia were analyzed. Hypotension was detected in 29 patients (43%) in cryoballoon AF ablation patients and in 30 patients (53%) in the EAM approach (*p* = 0.43). Regarding respiratory endpoints, there was no difference in hypercapnia and hypoxic episodes. Hypercapnia was detected in two patients per group, hypoxic episodes occurred in 24 patients (35%) in the cryoballoon AF ablation group and 19 patients (33%) in the EAM group. In line with this, cumulative time of hypoxic episodes was similar in both groups (cryoballoon: 24 (35%) vs. EAM: 19 (33%), *p* = 0.97). Detailed information on respiratory and hemodynamic safety is provided in Table 3.

**Table 3:** Respiratory and hemodynamic safety endpoints

	Total	Cryoballoon PVI	EAM	*p* value
	125	68	57	
MAP < 65 mmHg, patients	59 (47)	29 (43)	30 (53)	0.43
Hypercapnia tpCO_2_ > 70 mmHg, patients	4 (3)	2 (3)	2 (4)	0.86
Hypoxia tSpO_2_ < 90%, patients	43 (34)	24 (35)	19 (33)	0.97
Cumulative hypoxia time, minutes	343	189	155	0.86

Values are *n*, *n* (%), mean ± SD

*EAM* electroanatomical mapping, *MAP* mean arterial pressure, *SD* standard deviation

## Discussion

Electrophysiological procedures are a rapidly growing field, technological advances accelerate the development of new ablation methods and alternatives [1, 13]. The sedation requirements vary depending on the ablation site, arrhythmia, methodological approach and patient comfort that is needed for ablation. EAM procedures require deep conscious sedation to provide a strictly immobilized patient to warrant catheter stability, avoid map shifts, procedure associated complications and arrhythmia recurrence [9, 14]. For the single-shot cryoballoon technique, immobilization is less essential and no differences in arrhythmia recurrence or complication rates were found comparing moderate sedation and general anesthesia [3, 6]. Common cardiovascular risk factors and associated diseases such as obesity, smoking, sleep apnea or chronic obstructive lung disease (COPD) affect the respiratory system and could affect sedation [15]. Impaired hemodynamics for instance by arrhythmia or heart failure are also a common sedation complicating factor. Patients undergoing an EAM approach or cryoballoon ablation showed no difference in sedation relevant respiratory, cardiovascular and hemodynamic risk factors of patients.

Maintenance of sedation is mainly achieved by continuous propofol administration. The sedation depth is determined mainly clinical and is checked repeatedly during the procedure. The amount of administered propofol is titrated clinically and has a high interindividual variability in metabolism. Propofol is mainly metabolized by the liver and up to one third by the kidneys [16]. Renal and hepatic function does not differ between groups in our cohort. The procedure duration in patients undergoing EAM procedures is significantly longer compared to patients undergoing cryoballoon procedures which is in line with previous reports [3]. To maintain sedation in an electroanatomical mapping procedure, a higher amount of propofol is needed compared to cryoballoon ablation. Propofol-related adverse events affect especially the cardiovascular and respiratory system [16]. Despite the higher propofol dose and longer procedure duration, blood pressure drops (MAP < 65 mmHg), hypercapnia (tpCO_2_ > 70 mmHg) and hypoxia (tSpO_2_ < 90%) did not occur more often in the EAM group compared to the cryoballoon group. One could speculate, that a longer procedure duration and accumulation of propofol would increase the risk for adverse events, but complex 3D-mapping procedures seem to be as safe as cryoballoon ablation procedures under conscious sedation. Comparing respiratory parameters throughout the procedures, absolute tpCO_2_ values are higher in cryoballoon patients compared to EAM patients. These findings are surprising, because as indicated above, EAM procedures require deeper sedation than cryoballoon procedures, making higher tpCO_2_ plausible. Nevertheless, our measured tpCO_2_ values indicate the opposite. One might speculate that increase of tpCO_2_ levels may be related to PV occlusion by the cryoballoon during ablation, leading to impaired pulmonary perfusion. Interestingly, studies evaluating this condition, show that capnography, evaluating end-tidal CO_2_ levels, is very sensitive to detect acute changes in pulmonary perfusion, especially during ablation of the superior PVs under general aneasthesia. However, capnography shows an immediate decrease of end-tidal CO_2_ partial-pressure during PV occlusion [17, 18]. An increase of end-tidal CO_2_ partial-pressure during cryoballoon occlusion has not been observed, possibly because CO_2_ diffusion is compensated by the lung areas drained by the remaining PVs. On the other hand, phrenic nerve stimulation leading to frequent right-sided diaphragmal contractions may serve as an explanation for an increase in tpCO_2_. Recording of continuous motor action potentials (CMAP) is an established technique to monitor phrenic nerve function during cryoballoon ablation at the septal pulmonary veins. A ventilator depressive effect of phrenic nerve stimulation appears comprehensible but has not been reported yet.

In contrast, EAM patients have a more depressed MAP in the course of the procedure compared to cryoballoon patients, probably indicating the anticipated deeper sedation with higher propofol doses and higher opioid requirement.

## Limitations

This is a non-randomized, prospective observational study comparing two heterogeneous groups regarding the index arrhythmia. However, baseline characteristics indicate that patients are overall well comparable.

## Conclusion

Despite longer procedure duration and deeper sedation requirement, conscious sedation in EAM procedures appears to be as safe as conscious sedation in cryoballoon AF ablation procedures regarding hemodynamic and respiratory safety endpoints.

## References

[1] Page RL, Joglar JA, Caldwell MA, Calkins H, Conti JB, Deal BJ, Estes NAM, Field ME, Goldberger Z, Hammill S (2015). ACC/AHA/HRS guideline for the management of adult patients with supraventricular tachycardia: a report of the American College of Cardiology/American Heart Association Task Force on Clinical Practice Guidelines and the Heart Rhythm Society. J Am Coll Cardiol.

[2] Kirchhof P, Benussi S, Kotecha D, Ahlsson A, Atar D, Casadei B, Castella M, Diener HC, Heidbuchel H, Hendriks J, Hindriks G, Manolis A, Oldgren J, Popescu B, Schotten U, Van Putte B, Vardas P, Agewall S, Camm J, Baron E, Budts W, Carerj S, CAsselma F, Coca A, De Caterina R, Deftereos S, Dobrev D, Ferro JM, Fillipatos D, Gorenek B, Guenoun M, Hohnloser S, Kolh P, Lip GYH, Manolis A, McMurray J, Ponikowski P, Rosenhek R, Ruschitzka F, Savelieva I, Sharma S, Suwalski P, Tamargo JL, Taylor CJ, Van Gelder I, Voors AA, Windecker S, Zamorano J, Zeppenfeld K (2016). ESC Guidelines for the management of atrial fibrillation developed in collaboration with EACTS. Europace.

[3] Kuck K-H, Brugada J, Fürnkranz A, Metzner A, Ouyang F, Chun KJ, Elvan A, Arentz T, Bestehorn K, Pocock SJ (2016). Cryoballoon or radiofrequency ablation for paroxysmal atrial fibrillation. N Engl J Med.

[4] Lü F, Lin J, Benditt DG (2013). Conscious sedation and anesthesia in the cardiac electrophysiology laboratory. J Cardiovasc Electrophysiol.

[5] Kottkamp H, Hindricks G, Eitel C, Müller K, Siedziako A, Koch J, Anastasiou-Nana M, Varounis C, Arya A, Sommer P, Gaspar T, Piorkowski C, Dagres N (2011). Deep sedation for catheter ablation of atrial fibrillation: a prospective study in 650 consecutive patients. J Cardiovasc Electrophysiol.

[6] Wasserlauf J, Knight BP, Li Z, Andrei A-C, Arora R, Chicos AB, Goldberger J, Kim SS, Lin AC, Verma N, Bohn MM, Passman RS (2016). Moderate sedation reduces lab time compared to general anesthesia during cryoballoon ablation for AF without compromising safety or long-term efficacy. Pacing Clin Electrophysiol.

[7] Sairaku A, Yoshida Y, Hirayama H, Nakano Y, Kondo N, Kihara Y (2014). Don’t move during ablation of atrial fibrillation!. Int J Cardiol.

[8] Gerstein NS, Young A, Schulman PM, Stecker EC, Jessel PM (2016). Sedation in the electrophysiology laboratory: a multidisciplinary review. J Am Heart Assoc.

[9] Thomas SP, Thakkar J, Kovoor P, Thiagalingam A, Ross DL (2014). Sedation for electrophysiological procedures. Pacing Clin Electrophysiol.

[10] Kuck K-H, Albenque J-P, Chun KJ, Fürnkranz A, Busch M, Elvan A, SClüter M, Braegelmann KM, Kueffer FJ, Hemingway L, Arentz T, Tondo C, Brugada J, FIRE AND ICE Investigators (2019). Repeat ablation for atrial fibrillation recurrence post cryoballoon or radiofrequency ablation in the FIRE AND ICE Trial. CircArrhythm Electrophysiol.

[11] Pott A, Kraft C, Stephan T, Petscher K, Rottbauer W, Dahme T (2018). Time-to-isolation guided titration of freeze duration in 3rd generation short-tip cryoballoon pulmonary vein isolation–Comparable clinical outcome and shorter procedure duration. Int J Cardiol.

[12] Pott A, Petscher K, Messemer M, Rottbauer W, Dahme T (2016). Increased rate of observed real-time pulmonary vein isolation with third-generation short-tip cryoballoon. J Interv Card Electrophysiol.

[13] Hosseini SM, Rozen G, Saleh A, Vaid J, Biton Y, Moazzami K, Heist EK, Mansour MC, Kaadan MI, Vargel M, Ruskin JN (2017). Catheter ablation for cardiac arrhythmias: utilization and in-hospital complications, 2000 to 2013. JACCClinElectrophysi.

[14] Di Biase L, Conti S, Mohanty P, Bai R, Sanchez J, Walton D, John A, Santangeli P, Elayi C, Beheiry S, Gallinghouse J, Mohanty S, Horton R, Biley S, Burkhardt D, Natal A (2011). General anesthesia reduces the prevalence of pulmonary vein reconnection during repeat ablation when compared with conscious sedation: results from a randomized study. Heart Rhythm.

[15] Apfelbaum JL, Gross JB, Connis RT, Agarkar M, Arnold DE, Coté CJ, Dutton R, Madias C, Nickinovich DG, Schwartz PJ, Tom JW, Towbin R, Tung A for the American Society of Anesthesiologists Committee on Standards and Practice Parameters (2018). Practice guidelines for moderate procedural sedation and analgesia 2018: A report by the American Society of Anesthesiologists Task Force on moderate procedural sedation and analgesia, the American Association of Oral and Maxillofacial Surgeons, American College of Radiology, American Dental Association, American Society of Dentist Anesthesiologists, and Society of Interventional Radiology. Anesthesiology.

[16] Sahinovic MM, Struys MMRF, Absalom AR (2018). Clinical pharmacokinetics and pharmacodynamics of propofol. ClinPharmacokinet.

[17] Hoyt RH, Lim H (2015). Capnographic observations during cryoballoon ablation of atrial fibrillation. J Innov Card Rhythm Manag.

[18] Pickett RA, Owens K, Landis P, Sara R, Lim HW (2005). Cryoballoon-to-pulmonary vein occlusion assessment via capnography technique: where does occlusion testing by end-tidal CO2 measurement “Fit” as a predictor of long-term efficacy?. J Atr Fibrillation.

